# Weighted and directed interactions in evolving large-scale epileptic brain networks

**DOI:** 10.1038/srep34824

**Published:** 2016-10-06

**Authors:** Henning Dickten, Stephan Porz, Christian E. Elger, Klaus Lehnertz

**Affiliations:** 1 Department of Epileptology, University of Bonn, Sigmund-Freud-Straße 25, 53105 Bonn, Germany; 2 Helmholtz-Institute for Radiation and Nuclear Physics, University of Bonn, Nussallee 14-16, 53115 Bonn, Germany; 3 Interdisciplinary Center for Complex Systems, University of Bonn, Brühler Straße 7, 53175 Bonn, Germany

## Abstract

Epilepsy can be regarded as a network phenomenon with functionally and/or structurally aberrant connections in the brain. Over the past years, concepts and methods from network theory substantially contributed to improve the characterization of structure and function of these epileptic networks and thus to advance understanding of the dynamical disease epilepsy. We extend this promising line of research and assess—with high spatial and temporal resolution and using complementary analysis approaches that capture different characteristics of the complex dynamics—both strength and direction of interactions in evolving large-scale epileptic brain networks of 35 patients that suffered from drug-resistant focal seizures with different anatomical onset locations. Despite this heterogeneity, we find that even during the seizure-free interval the seizure onset zone is a brain region that, when averaged over time, exerts strongest directed influences over other brain regions being part of a large-scale network. This crucial role, however, manifested by averaging on the population-sample level only – in more than one third of patients, strongest directed interactions can be observed between brain regions far off the seizure onset zone. This may guide new developments for individualized diagnosis, treatment and control.

Epilepsy—one of the most prevalent neurological conditions with about 65 million affected individuals worldwide^1^—is nowadays conceptualized as a *network disease* with functionally and/or structurally aberrant connections on virtually all spatial scales^2^,^3^. According to this concept, a large-scale epileptic network comprises cortical and subcortical areas that generate and sustain normal, physiological brain dynamics during the seizure-free interval and are involved in the generation, maintenance, spread, and termination of pathophysiological activities such as seizures (both primary generalized and with a focal onset). An improved understanding of how this complex behavior—as seen, e.g., on the electroencephalogram (EEG)—emerges from human brain networks can probably be achieved with analysis approaches that take into account the interplay between the dynamic properties of brain regions and the network structures connecting them.

A network (or graph) is composed of a set of nodes and a set of edges, connecting the nodes^4^. Nodes in a large-scale epileptic network are usually assumed to represent distinct brain regions (i.e., local networks of neurons) and edges represent weighted and/or directed interactions between them (irrespective of their physical connectedness), and these nodes and edges constitute a functional network. Edges may change on various timescales, depending on physiological and pathophysiological conditions, and time-dependent edge properties are usually assessed with bivariate analysis techniques designed to characterize various linear or nonlinear aspects of strength and direction of an interaction^5^,^6^,^7^,^8^. With such techniques, particularly the so called seizure onset zone (SOZ)—the region of the brain from which initial seizure discharges can be recorded^9^—has been repeatedly characterized as a network region with distinctly increased strengths of intrinsic interactions^10^,^11^,^12^,^13^,^14^,^15^,^16^,^17^,^18^,^19^ and as a network region that appears to drive (in terms of a driver–responder relationship) other regions^20^,^21^,^22^,^23^,^24^,^25^,^26^,^27^,^28^,^29^,^30^.

Although these findings suggest the network architecture to be quite robust, thereby underlining the crucial role of the SOZ and its interactions within the epileptic network, their contribution to the understanding of the long-term dynamics of the epileptic process in such large-scale networks (spanning lobes and hemispheres) may be limited due to a number of methodological and conceptual issues: The majority of previous studies only assessed properties of brain interactions using EEG data from selected recording sites and only investigated selected epochs of pathophysiological activities (such as seizures and/or epileptiform discharges). Selections were mostly based on various cortical zones as used in the presurgical evaluation^9^, but it is not yet fully clear, if and how the various zones and their dynamics match the modern concepts of a large-scale functional epileptic network. A spatially and temporally confined a priori assignment of parts of the network to these zones might potentially bias analyses and results. Moreover, properties of interactions in functional epileptic networks may be influenced by processes acting on various time-scales, ranging from minutes to days^31^,^32^,^33^.

In addition, different analysis techniques—that are based on different concepts and that may be affected differently by various aspects of the recording—were used to characterize either aspects of the strength or of the direction of an interaction. Concerning the latter, quite often has an estimator’s modulus been interpreted as some strength of an interaction (the sign indicates the direction), which might not generally be valid and can lead to severe misinterpretations particularly for uncoupled and strongly coupled systems^34^,^35^,^36^. Such strong couplings can quite often be observed with pathophysiologic synchronization phenomena, such as epileptic seizures^7^.

We here present an analysis strategy that addresses these conceptual and methodological open issues and allows one to characterize interactions in evolving large-scale functional epileptic networks without a priori restrictions. We perform a temporally highly resolved and combined characterization of both weighted and directed interactions—evolving over days to weeks—between all sampled regions in the brains of 35 patients that suffered from drug-resistant focal seizures with different anatomical onset locations. We divide the individually sampled brain regions into different categories (focal (*f*), neighborhood (*n*), and other (*o*)), which allows us to perform analyses on the population-sample level and compare findings with respect to the SOZ with those achieved in previous studies.

Given the by now commonly accepted strong evidence for synchronization to play an important role in brain functioning and dysfunctioning^7^,^37^,^38^, we employ analysis techniques that are based on the most prominent concepts of synchronization, namely phase synchronization and generalized synchronization^39^, and use a phase-dynamics-based approach (PA) and an information-content-based approach (IA) to characterize strength and direction of network edges from multi-channel, multi-day, intracranial electroencephalographic (iEEG) recordings. PA consists of estimating the strength of an interaction with the mean phase coherence *R*^40^ and the direction of an interaction with the directionality index *d* that is based on the evolution map approach^41^. IA consists of estimating the strength of an interaction with the order parameter *γ*^42^ and the direction of an interaction with directionality index *T* that is based on symbolic transfer entropy^43^. Despite their conceptional and methodological differences, both estimators for the strength of an interaction assign a low/high weight to an edge that connects weakly/strongly coupled, interacting brain regions. Similarly, both estimators for the direction of an interaction assign a directionality to an edge that indicates either a driving, a responding, or an uncoupled or symmetric bidirectional relationship between brain regions. This assignment, however, incorporates the actual coupling strength (as assessed with the respective estimator for the strength of an interaction) to avoid misinterpretations about directionality in regimes of zero and strong couplings.

## Results

**Figure 1:**
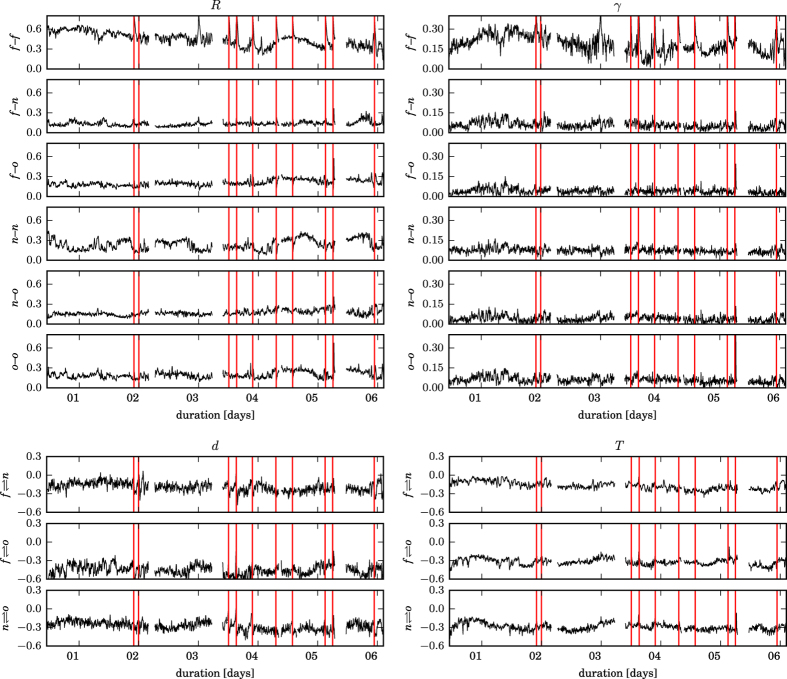
Exemplary long-term variations of weighted and directed interactions in the epileptic brain. Temporal evolutions of the strength (estimated with *R* and *γ*; upper six rows) and the direction of interactions (estimated with *d* and *T*; lower three rows) derived from multi-channel, multi-day iEEG data from one patient. Ten spontaneous seizures occurred during the recording (vertical red lines mark electrical onset of seizures). Data are from interactions within the SOZ (*f*–*f*), between SOZ and neighborhood (*f*–*n*), between SOZ and other brain areas (*f*–*o*), within neighborhood (*n*–*n*), between neighborhood and other brain areas (*n*–*o*), and within other brain areas (*o*–*o*). The direction of interaction 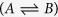 is encoded in the sign of the respective estimate *d* and *T*: if negative, then *B drives A (B* → *A*); if positive, then *A drives B (A* → *B*). We here consider the direction of interactions between but not within brain regions. All time profiles were smoothed using a sliding Hamming-window of length 5 min for better legibility, and discontinuities are due to recording gaps. Tics on x-axes denote midnight.

In Fig. 1, we show exemplary temporal evolutions of the strength and the direction of interactions between various brain regions derived from an iEEG recording that lasted about six days. Highest strengths of interactions were confined to the seizure onset zone (SOZ; *f*–*f*) and they tended to decrease with an increasing distance to the SOZ. *R* and *γ* rated the average strength of interactions within the neighborhood (*n*–*n*) and within remote brain regions (*o*–*o*) differently. With both indices, *d* and *T*, we observed pronounced directed interactions between the SOZ as well as its neighborhood and remote brain regions (*f*–*o* and *n*–*o*). Throughout the recording, remote brain regions tended to drive both the SOZ (*o* → *f*) and its neighborhood (*o* → *n*). Only towards the end of the recording period became the driving of the SOZ by its neighborhood (*n* → *f*) more distinct.

**Figure 2:**
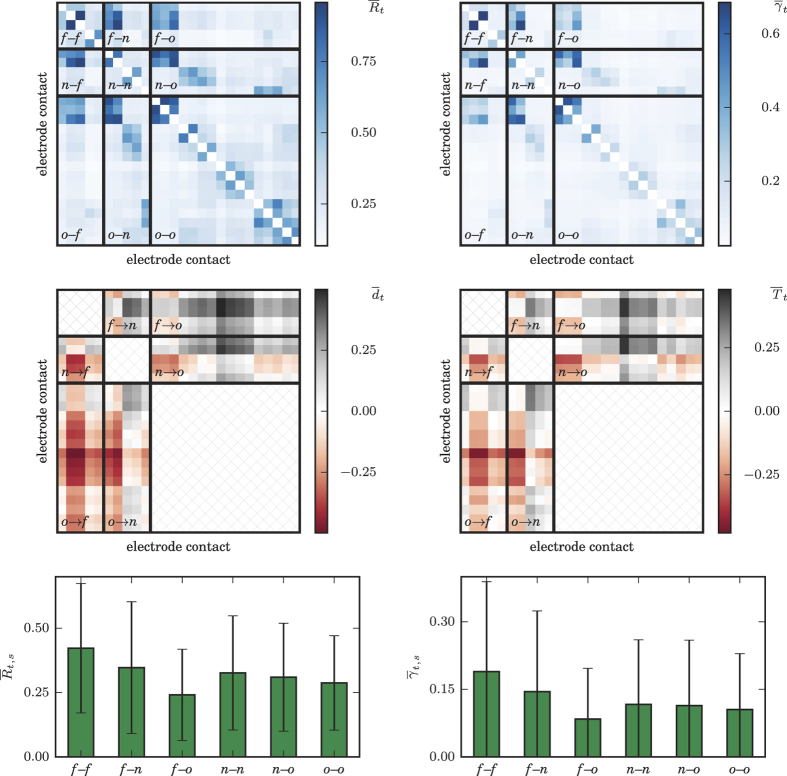
Exemplary temporal and spatial averages of weighted and directed interactions. Temporal average of strength (upper row: 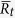, 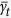) and direction (middle row: 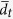, 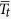) of all pair-wise interactions estimated from a multi-channel, multi-day iEEG recordings from one patient (cf. Fig. 1). Thick black lines delineate categorized brain regions; direction of interactions within brain regions were not considered (cross-hatched matrix entries). Positive (negative) values of estimators for the direction of interactions indicate that sites listed on the ordinate drive (are being driven by) sites listed on the abscissa. Temporally and spatially averaged strengths (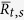, 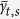) for all interactions within and between brain regions are shown in the lower row. Errorbars indicate the standard deviation over space and time.

All time profiles exhibited a more or less pronounced variability that appeared to depend on which brain regions are interacting. This variability did not appear to coincide with seizure-related activities. Having said this, some profiles exhibited some temporal structure, which seemed to be partly periodic, indicating a possible influence of daily rhythms, and/or of changes of the anticonvulsant medication. Overall, the temporal variabilities of both strength and direction of interactions as assessed with the PA (*R, d*) were comparable to those obtained with IA (*γ, T*), and the same was true for the spatially resolved temporal averages of indices (cf. Fig. 2). Nevertheless, *R* and *T* in general appeared to provide a higher contrast than their counterparts.

### Population-sample level statistics of strength of interactions between brain regions

**Figure 3:**
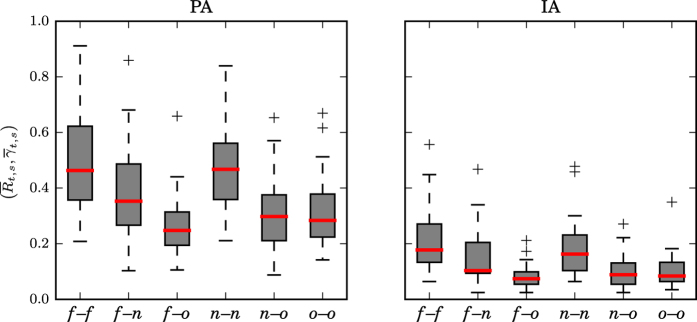
Distribution of temporally and spatially averaged strengths of interactions within and between brain regions from all patients. Strength of interactions obtained with PA (left) and with IA (right). Interactions within the SOZ (*f*–*f*), between SOZ and its neighborhood (*f*–*n*), between SOZ and other brain areas (*f*–*o*), neighborhood–neighborhood interactions (*n*–*n*), as well as neighborhood–other (*n*–*o*) and other–other interactions (*o*–*o*). Bottom and top of a box are the first and third quartiles, and the (red) band inside a box is the median. The highest and lowest occurring value within the 1.5-fold interquartile range are drawn as bar of the whiskers, and outliers are plotted as individual + signs.

In order to demonstrate extendability of these observations beyond exemplary data, we estimated—for all patients and using their corresponding full datasets—statistical properties of the distributions of the temporal and spatial means (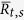 and 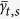) of the strength of all interactions within and between brain regions (cf. Fig. 3) and evaluated possible differences between distributions. On the population-sample level, we observed highest median values for interactions within the SOZ (*f*–*f*), for neighborhood–neighborhood interactions (*n*–*n*), and for interactions between SOZ and its neighborhood (*f*–*n*). The strengths of these interactions, however, did not differ significantly (Mann–Whitney *U* test), neither with PA nor with IA, which could be related to the high interindividual variability. When compared to the aforementioned, solely short-range interactions within brain regions, we observed (with both approaches) clearly decreased strengths only for long-range interactions between SOZ and other brain areas (*f*–*o; p* < 0.005, Mann–Whitney *U* test) as well as for neighborhood–other (*n*–*o; p* < 0.05, Mann–Whitney *U* test) and other–other interactions (*o*–*o; p* < 0.005, Mann–Whitney *U* test).

Table 1 lists the number (percentage) of patients that presented with highest strengths (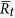, 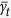) in the respective interaction between brain regions, taking into account the high interindividual variability of the number of pairs of electrode contacts within each category (cf. Table 3). In about 65% of patients, strongest interactions were confined to the SOZ (*f*–*f*) and in 24% to its neighborhood (*n*–*n*); these interactions were mostly short-ranged. Another 35% of patients presented with strongest interactions between brain regions far off the SOZ (*o*–*o*), comprising short-, medium-, and long-range interactions. The heterogeneity of seizure onset locations had no influence on these observations.

On a population-sample level, we attained findings similar to all aforementioned ones when restricting analyses to data from the subsets *day, night, inter-ictal*, or *pre-ictal* (see Methods), which might indicate strength of interactions to be largely independent of physiological and pathophysiological activities.

### Population-sample level statistics of direction of interactions between brain regions

**Figure 4:**
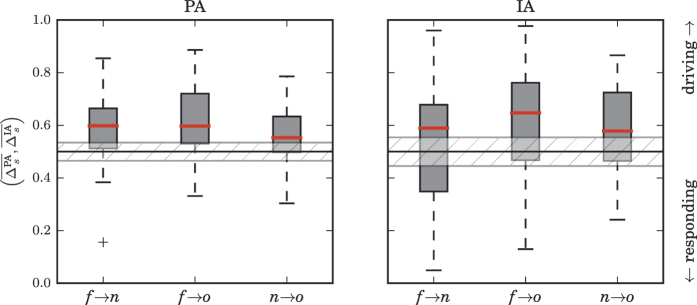
Distribution of spatially averaged rates of a specific directionality indication 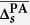 (left) and 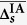 (right) from all patients. Interactions between SOZ and neighborhood 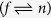, between SOZ and other brain areas 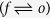, and between neighborhood and other brain areas 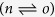. The direction of interaction 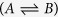 is encoded in averaged rates of a specific directionality indication: if 
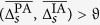
, then *A drives B* more frequently (*A* → *B*); if 
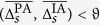
, then *A responds* to *B (A* ← *B*) more frequently. *ϑ* indicates an equal rate of driving and responding indications (black line). The gray-hatched area indicates no *predominant* direction (see section Methods). Description of boxes and symbols as in Fig. 3.

Statistical properties of the distributions of the spatially averaged rates of a specific directionality indication 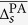 and 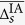 of all interactions between brain regions are shown for the population-sample level in Fig. 4. We observed median rates to exceed the threshold ϑ of an equal rate of driving and responding indications (see section Methods) for each interaction, which points to mainly driving interactions in the majority of patients (region *A* drives region *B* most of the time). Directionality indications of these interactions, however, did not differ significantly (Mann–Whitney *U* test), neither with PA nor with IA, and, in general, 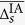 indicated a higher interindividual variability than 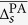. Nevertheless, both the SOZ, and its neighborhood, appeared to *predominantly* drive 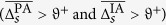 its neighborhood (*f* → *n*) as well as other brain regions (*f* → *o*) in about 60% of patients (see section Methods for a definition of the range of rates [*ϑ*^−^, *ϑ*^+^] above/below which we observe predominant driving/responding). Noticeable differences between approaches could be observed for interactions between the SOZ and other brain regions: With IA we observed the SOZ to be predominantly driven 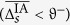 by its neighborhood in 33% of patients, as opposed to 20% of patients observed with PA (note that the data of one of these two patients must be regarded as outlier, see Fig. 4).

In Table 2, we list the number (percentage) of patients, for which highest rates of a specific directionality indication Δ^PA^ or Δ^IA^ for a given interaction between brain regions could be observed significantly more often than by chance (hypergeometric test). In about two third (using PA) to about one half (using IA) of patients, our analyses indicated the SOZ to predominantly drive other brain regions (*f* → *o*). In another one third of patients, the neighborhood of the SOZ presented as a predominant driver of other brain regions (*n* → *o*). Other directed interactions and particularly those indicating a predominantly responding of the SOZ were seen in 7–17% of patients only. We note that all the aforementioned indications for a specific direction of interaction were quite stable over time, since the highest rates Δ^PA^ that entered analyses clearly exceeded 0.61. Stability was even more pronounced (Δ^IA^ > 0.91) when characterizing the direction of interaction with IA. The heterogeneity of seizure onset locations had no influence on these observations.

As with our observations for the strength of interactions, we attained similar findings when restricting analyses to data from the subsets *day, night, inter-ictal*, or *pre-ictal*, which—on the population-sample level—might indicate also direction of interactions to be largely independent of physiological and pathophysiological activities.

### Correlations between approaches

**Figure 5:**
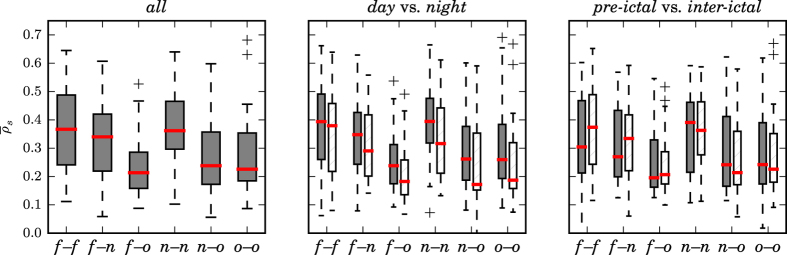
Distribution of spatially averaged Pearson product-moment correlation coefficients 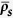 between estimators *R* and *γ* for the strength of interactions within and between brain regions. Correlation coefficients obtained from full dataset (left), from subsets *day* (filled bars) and *night* (hatched bars) (middle), and from subsets *pre-ictal* (filled bars) and *inter-ictal* (hatched bars) (right). Interactions as well as description of boxes and symbols as in Fig. 3.

**Figure 6:**
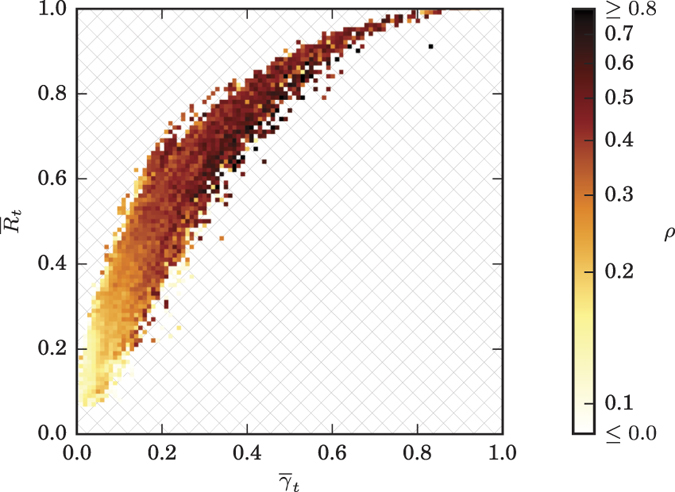
Relationship between PA- and IA-based estimators for the strength of interactions. Scatter plot of population-sample level averages of Pearson product-moment correlation coefficients *ρ* between estimators *R* and *γ* as a function of the particular temporally averaged strength of interactions for all pairwise interactions (findings based on full datasets). Negative correlations could only be observed rarely and for 
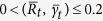
; these were clipped.

So far, our findings indicate that on the population-sample level PA- and IA-based estimators appear to provide comparable information about strength and direction of interactions in large-scale epileptic brain networks. However, as evidenced in the left part of Fig. 5, correlations between estimators for the strength of interactions attained median values between 0.2 and 0.35 only, with highest values confined to the interactions *f*–*f, n*–*n*, and *n*–*o*. Again, interindividual variability was quite high, with values ranging between 0.05 and 0.7. Note that variabilities between interactions were rather small compared to interindividual variabilities, and none of these variabilities appeared to reflect the high variability of the anatomical onset locations of seizures or of number and anatomical locations of intracranial electrodes. When restricting correlation analyses to data from the subsets (*day, night, inter-ictal*, or *pre-ictal*), overall findings on a population-sample level were quite similar to those seen for the full dataset (cf. Fig. 5). For the subset *night*, however, median correlations slightly decreased (as compared to those from subset *day*) for all interactions but the within-SOZ (*f*–*f*) interaction (middle part of Fig. 5). This might reflect the dependence on daily rhythms seen for both estimators for strength of interactions. On a population-sample level, differences between findings from subset *pre-ictal* and *inter-ictal* were negligible (right part of Fig. 5), and in up to four patients correlations were negative when restricting data to subset *pre-ictal*. Similar to our findings for the strength of interactions (see Fig. 3), the median level of correlations tended to decrease with increased distance to the SOZ. This might be explained by higher correlations being predominantly related to pairs of higher temporal means of strength of interactions 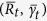 (cf. Fig. 6).

Investigating the relative amount of time 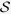 for which both approaches, PA and IA, indicated the same directionality (driving, responding, or bidirectional), we obtained a median value of 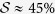 for each interaction between brain regions on a population-sample level (data not shown). Interindividual variabilities were highest for interactions between the SOZ and its neighborhood 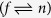, exceeding those for other interactions by a factor of about 2. As expected, physiological and pathophysiological activities did not affect the dissimilarity between approaches.

Summarizing these findings, the investigated approaches provide largely independent, non-redundant information about the strength and the direction of interactions.

## Discussion and Conclusion

We investigated weighted and directed interactions in evolving, large-scale, functional epileptic networks using analysis approaches that are based on either the phase-dynamics (PA) or on the information-content (IA) of brain dynamics. We derived such networks with high temporal resolution from multi-day, multi-channel intracranial EEG (iEEG) recordings from 35 epilepsy patients that suffered from pharmacoresistant focal seizures with different anatomical onset locations. Characterizing strength and direction of interactions between all sampled brain regions (nodes) in a combined manner to avoid spurious inference about underlying driving structures, we aimed at (i) identifying network nodes that can be delineated through their interaction properties, (ii) characterizing the possible impact of various physiological and pathophysiological activities on interaction properties, and (iii) quantifying similarities/dissimilarities between analysis approaches.

Despite conceptual and methodological differences between approaches, both identified the seizure onset zone (SOZ) as a brain region whose interaction properties differ from those of other sampled brain regions in the majority of patients, even during the seizure-free interval and despite the heterogeneity of seizure onset locations. On the population-sample level, strengths of interactions were highest within the SOZ and declined gradually with an increasing distance to the SOZ. In about half the cases, the SOZ appeared to predominantly drive its neighborhood as well as other brain regions. These findings were largely independent of physiological (daily rhythms) and pathophysiological activities (pre-ictal vs. inter-ictal states) and were not affected by the different sensor types used to collect iEEG data (we repeated all analyses for subgroups of patients, whose seizure onset zones were sampled with the same sensor type and obtained qualitatively similar findings; data not shown).

As regards the strength of interactions, our findings concerning its level and spatial distribution are in line with previous studies^10^,^11^,^12^,^13^,^14^,^15^,^16^,^17^,^18^,^19^ that underline the crucial role of the SOZ and its interactions within the epileptic brain. However, our investigations also revealed comparably strong or even stronger interactions between brain regions far off the SOZ, as identified in about one third of patients; the crucial role of the SOZ manifested by averaging on the population-sample level only. The importance of brain regions well outside the SOZ has been emphasized repeatedly^7^,^31^,^44^ as they are part of an epileptic network and contribute to ictogenesis^45^. Together with these findings, our observations put into perspective previous studies that proposed estimation of the strength of interaction as a means to identify and localize the epileptogenic zone in the presurgical evaluation of pharmacoresistant epilepsies^10^,^11^,^13^,^14^,^17^,^46^. These contradicting observations may be related to differences in recording durations (multi-day vs. selected samples lasting few minutes to few hours), the extent of spatial sampling, as well as analysis techniques.

As regards the directions of interactions, these had so far been studied in focal epilepsies mostly during seizures^20^,^21^,^22^,^23^,^24^,^25^,^26^,^27^,^30^,^47^,^48^ or for selected periods with pronounced interictal epileptiform activities^21^,^27^,^28^,^29^. In the majority of these studies, analysis techniques based on the concept of Granger causality had been employed, and findings consistently point to the SOZ as a brain region that exerts the strongest influence on other brain regions. Such a *focal driving*^49^, however, might be spuriously induced if the actual strength of an interaction is not taken into account^34^,^35^,^36^. Our combined assessment of strength and direction of interactions explicitly considers this shortcoming, thus allowed us to effectively differentiate between various coupling regimes—which are to be expected in evolving epileptic brain networks^7^,^3^,^36^—, and to minimize the risk for misinterpretations. Although our findings also point to a predominant focal driving, which can even be observed over long periods during the seizure-free interval, this was the case for about 50% of the patients only. It remains to be shown whether the driving of the SOZ by its neighborhood or even by remote brain regions (observed in particular with IA) reflects an effective *inhibitory surround*^50^,^51^ that prevents the spreading of epileptiform activities during the seizure-free interval.

We observed both, averaged strength and direction of interactions to be largely independent of physiological and pathophysiological activities on a population-sample level. Interactions in the epileptic brain, however, can be affected by changes in the antiepileptic medication^52^,^53^,^54^ or by the state of vigilance^55^, and recent studies showed characteristics of functional epileptic networks to fluctuate on various temporal scales^31^,^32^,^33^. In line with these studies, we observed that the temporal variability seen for all estimators for the strength and direction of interactions was quite high (data not shown here), both intra- and interindividually. We therefore speculate that the observed independence and with it, a reduced traceability of specific phenomena may be caused by the applied averaging procedures.

The applied analysis approaches (PA and IA) capture different characteristics of the complex interaction dynamics in large-scale epileptic networks^7^,^12^,^56^. While phase-dynamic-based estimators are hardly influenced by changes in amplitudes, information-content-based estimators explicitly take into account changes in both phase and amplitude to measure the flow of information between interacting brain regions. Hence it is hardly surprising that we observed relationships between estimators for the strength of interactions to range between uncorrelated and highly correlated. Also, congruence between estimators for the direction of interaction varied considerably, both intra- and interindividually, and in – on average – 40% of time, one approach indicated some directionality while the other indicated either a bidirectional interaction or no interaction at all. This high variability led us to conclude, that the investigated approaches provide largely independent, non-redundant information about the strength and the direction of interactions. Nevertheless, on a population-sample level and despite the heterogeneity of investigated cases, PA- and IA-based estimators appear to provide comparable information about strength and direction of interactions in epileptic brain networks, and it remains to be investigated whether this was caused by the applied averaging procedures. Interestingly, on a population-sample level correlations between approaches were highest for interactions between SOZ and its surroundings. This might indicate that both approaches appear to be well suited to characterize the interaction dynamics of the SOZ. However, for its (functional) delineation from other brain areas a combination of multiple approaches would be advisable.

Our investigations showed that the PA estimator for the strength and the IA estimator for the direction of interactions in general appeared to provide a higher contrast than their counterparts. This might reflect different sensitivities of approaches for the various types of synchronization phenomena^7^,^39^ that may underly physiological and pathophysiologic functioning in large-scale brain networks. Whether this also implies a higher sensitivity and specificity for interactions in the epileptic brain, needs to be investigated, e.g. by making use of surrogate techniques^36^,^57^, notwithstanding urgently needed improvements in tests for the significance of the respective estimators.

To conclude, our spatially and temporally resolved investigations of weighted and directed interactions in the epileptic brain using complementary analysis approaches indicate the seizure onset zone as a node that exerts strongest directed influences over other brain regions being part of a large-scale functional network. While this was a consistent finding on an averaged population-sample level, our investigations also revealed a high interindividual variability, which points to not yet fully understood influences of various physiological and pathophysiological activities. Together with recent advances in the data-driven characterization of interaction properties (e.g., through approaches for delayed directed interactions^58^,^59^ or through multivariate extensions of approaches^60^,^61^,^62^), we expect an improved characterization of evolving epileptic brain networks, which may help to advance diagnosis and to develop new therapeutic strategies.

## Methods

### Patient characteristics

The 35 patients (18 women, 17 men; mean age at onset of epilepsy 12.9 yrs, range 0–41 yrs; mean duration of epilepsy 23.0 yrs, range 1–56 yrs) included in this retrospective study suffered from pharmacoresistant focal seizures with different anatomical onset locations (23 patients with left and 12 patients with right hemispheric onsets), which required prolonged invasive monitoring with intrahippocampal depth electrodes and subdural grid- and strip-electrodes. Decisions regarding electrode placement were purely clinically driven and were made independently of this study. Patients received different antiepileptic drugs (AEDs) with different mechanisms of action, and the majority of patients were under combination therapy with two or more AEDs. During presurgical evaluation AEDs were reduced in a patient-specific manner, and many patients did not have discontinuation of all AEDs. All patients signed informed consent that their clinical data might be used and published for research purposes and are seizure free post-operatively (Engel class 1A^63^). The study protocol had previously been approved by the ethics committee of the University of Bonn, and methods were carried out in accordance with the approved guidelines.

### Intracranial EEG Recordings

Multi-channel, multi-day intracranial electroencephalographic (iEEG) data were recorded with, on average, 51 electrode contacts (range: 14–88). The mean recording duration amounted to 114 h (range: 18–324 h) and, on average, five seizures were captured per patient (range: 0–24; total: 173). We band-pass-filtered iEEG signals between 1–45 Hz and suppressed possible contributions of the power line frequency using a notch filter. We sampled the data at 200 Hz (sampling interval Δ*t* = 5 ms) using a 16 bit analog-to-digital converter, and referenced the signals against the average of two electrode contacts outside the focal region. Reference contacts were chosen individually for each patient.

The time of seizure onset was visually identified on the iEEG as the time of earliest clear change from the patient’s baseline or normal background iEEG that eventually led to an electrographic seizure. Subclinical seizures were neglected in our analyses. In 15 patients, seizure onset was confined to the mesial-temporal structures, in eight patients to the polar or lateral aspects of the temporal lobe, and in seven patients to the frontal lobe. In five patients, the seizure onset zone could be identified in either the insula or the parietal or occipital lobe.

We assigned electrode contacts to three categories^45^. Category *f (focal*) comprised all contacts located within the clinically identified seizure onset zone (SOZ; those contacts where first ictal discharges were recorded^9^; on average, 9 contacts/patient (range: 1–30); 19.2% of all contacts). Category *n (neighborhood*) comprised those contacts not more than two contacts distant to those from *f* (on average, 4 contacts/patient (range: 0–18); 8.4% of all contacts). Note that a neighborhood of the SOZ could be defined in 30 of the 35 patients. All remaining contacts were assigned to category *o (other*; on average, 39 contacts/patient (range: 9–84); 72.4% of all contacts). Table 3 reports the mean number of contact pairs for interactions within and between brain regions.

### Estimating strength and direction of interactions

We employed two popular analysis approaches that are based on either the phase-dynamics (PA) or on the information-content (IA) of iEEG time series. In the following, we denote with *x*_*j*_ := *x*(*j*Δ*t*) and *y*_*j*_ := *y*(*j*Δ*t*), *j* = 1, …, *N*, two iEEG time series recorded at brain sites *X* and *Y*, and *N* is the number of data points per time series.

#### Approaches based on phase-dynamics (PA)

Widely used phase-dynamics-based estimators for the strength and the direction of interactions are the mean phase coherence *R*^40^ and the directionality index *d* derived from the evolutionary map approach^41^. Both estimators require phase time series *ϕ*^*X*^ (*j*) and *ϕ*^*Y*^ (*j*), *j* = 1, …, *N*, that we derived from the iEEG time series *x*_*j*_ and *y*_*j*_ using the analytic signal approach^64^ with which instantaneous phases are obtained from the Hilbert transform of a time series. An important property of this approach is that the instantaneous frequency—in case of two or more superimposed oscillatory components—relates to the predominant frequency in the Fourier spectrum^65^,^66^. The predominant frequency may be subject to fluctuations in the time series, in which case the instantaneous frequency varies rhythmically around the predominant frequency resulting in spurious estimates of the instantaneous phase. Such effects can nevertheless be reduced, e.g., by taking the temporal average (cf. Eq. 1). We note that—from an electrophysiological point of view—it might be more reasonable to look adaptively (via the Hilbert transform) at interactions between predominant rhythms in the iEEG than to look at interactions in some a priori fixed frequency bands (e.g., via wavelet) for which there is no power in the time series^12^,^66^.

Phase synchronization between noisy (and even chaotic) systems is usually defined via the phase entrainment condition |*μϕ*^*X*^ (*j*) − *νϕ*^*Y*^ (*j*)| ≤ const., where *μ* and *ν* are integers, and the equal sign holds for the case of phase locking. We here restrict ourselves to the case *μ* = *ν* = 1, and estimate the degree of synchronization from the circular distribution of the measured phase differences using the mean phase coherence:


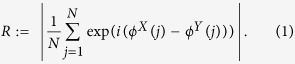


*R* approaches zero, if the systems are uncoupled, and for fully synchronized systems, *R* → 1. *R* increases monotonically with an increasing coupling strength and thus serves as an estimator for the strength of an interaction.

In order to estimate the direction of an interaction, we derive the directionality index *d* from the phase time series. For some fixed time *δ*, the phase increments 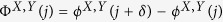 are assumed to be generated by a two-dimensional noisy map 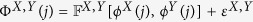, where the last term represents noisy perturbations. The map 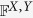 can be approximated using finite Fourier series 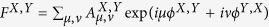. We include all Fourier terms, which fulfill a combination of summation indices: |*μ*| ≤ 3 for |*ν*| = 0 and |*ν*| ≤ 3 for |*ν*| = 0 and *μ* = *ν* = 1^41^,^67^. Given *F*^*X*,*Y*^, the mutual influence of the systems can be quantified with:


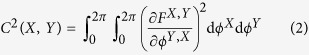


and the directionality index, which serves as an estimator for the direction of an interaction, can be defined as:


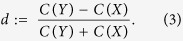


*d* is confined to the interval [−1, 1]. For *d* → 1, *X* predominantly drives *Y*, and in the case of negative values, the direction of driving is reversed. If *d* approaches zero, the coupling is bidirectional.

#### Approaches based on information-content (IA)

As information-theoretic estimators for the strength and the direction of interactions we here use the order parameter *γ*^42^ and the directionality index *T* that is based on symbolic transfer entropy^43^. Both estimators require symbol time series 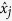 and 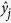 that we derived from the iEEG time series *x*_*j*_ and *y*_*j*_ using a technique of symbolization, which is based on a permutation of the amplitude values of a time series^68^. Let *l* and *m* denote the lag and embedding dimension, which have to be chosen appropriately for symbolization (e.g., by making use of embedding theorems and by choosing *l* in the order of the first zero-crossing of the autocorrelation function to achieve (at least linearly) independent state-space vectors). Then *m* amplitude values *s*_*j*_ := (*x*_*j*_, *x*_*j*+1_, …, *x*_*j*+1(*m*−*j*)_) for a given, but arbitrary time index *j* are arranged in ascending order 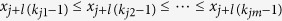 with rank *k*_*ji*_ and 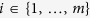. Equal amplitude values are arranged by their time index such that *k*_*j*1_ < *k*_*j*2_ if 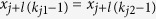. This ensures that every *s*_*j*_ is uniquely mapped onto one of the *m*! possible permutations, and a permutation symbol is defined as 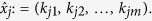 Relative frequencies of symbols provide an estimator for (joint and conditional) probabilities of the sequences of permutation indices.

In order to derive an estimator for the strength of an interaction, we assesses the consistency of changing tendencies of temporal permutation entropies 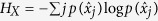 of symbol time series by splitting the time series into *η* = 1, …, *N*_*η*_ overlapping windows *w*_*η*_ with *N*_*w*_ data points each. The tendency can be quantified with the coefficient *S*_*X*_ (*w*_*η*_), which attains a value of 1 if *H*_*X*_ (*w*_*η*+1_) > *H*_*X*_ (*w*_*η*_), and −1 otherwise. The permutation entropies *H*_*Y*_ (*w*_*η*_) and the coefficient *S*_*Y*_ (*η*) are defined in complete analogy. The in-step behavior of *H*_*X*_ and *H*_*Y*_ will be identical if systems *X* and *Y* are fully coupled, and order parameter *γ*, as a measure for the strength of an interaction, then reads:


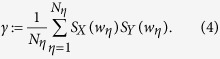


*γ* will be close to unity for fully coupled systems *X* and *Y* and zero for independent ones. Note that *γ* might attain slightly negative values. *γ* increases monotonically with an increasing coupling strength and thus serves as an estimator for the strength of an interaction.

In order to estimate the direction of an interaction, we derive the directionality index *T* from the symbolic transfer entropies^43^ of symbol time series 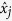 and 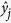. If systems *X* and *Y* are independent (uncoupled), the generalized Markov property *p*(*y*_*i*_|*y*_*i*−1_, *x*_*i*−1_) = *p*(*y*_*i*_|*y*_*i*−1_) (with the conditional transition probability density *p*(·|·)) holds, i.e., including previous states *x*_*i*−1_ and *y*_*i*−1_ does not improve the prediction of state *y*_*i*_. Deviations from this assumption can be quantified with the symbolic transfer entropy


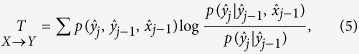


which measures the flow of information between symbol time series 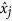 and 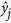. *T*_*Y*→*X*_ is defined in complete analogy, and the sum in equation (5) runs over all symbols. The directionality index


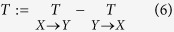


allows one to quantify the preferred direction of information flow. For *T* > 0, the predominant direction of flow of information is from system *X* to *Y (X* predominantly drives *Y*), and vice versa. For symmetric bidirectional couplings we expect *T* = 0. Note that in contrast to the definition of the phase-dynamics-based directionality estimator, we do not normalize *T* since the symbolic transfer entropies *T*_*X*→*Y*_ and *T*_*Y*→*X*_ are unbounded. Following previous work^69^, we chose *m* = 5 and *l* = 3 for symbolization and estimated *γ* with *N*_*w*_ = 2048 and *N*_*η*_ = 204.

#### Time-resolved analysis and down-streaming processing steps

We used a sliding-window approach with non-overlapping windows of *N* = 4096 data points (duration: 20.48 s) each to calculate the aforementioned estimators for the strength (*R* and *γ*) and direction of interactions (*d* and *T*) in a time-resolved manner for every possible combination of pairs of electrode contacts (for the sake of readability, in the following we omit the dependence of the window number for each estimator). We regard the choice of *N* = 4096 data points as a compromise between the required statistical accuracy for the calculation of estimators and approximate stationarity within a window length.

Based on previous investigations that employed bivariate time series analysis techniques to characterize strength and direction of interactions in studies on coupled driver–responder model systems^34^,^35^,^36^,^43^, we can expect strength and direction of an interaction to depend on some (hypothetical) coupling strength as follows:

- estimators for the strength of interactions increase monotonously with an increasing coupling strength until they reach their upper bounds for fully synchronized systems;
- estimators for the direction of interactions will attain non-zero values for some intermediate coupling strengths only;
- estimators for the direction of interactions will attain zero values for uncoupled (zero coupling strength) and for fully synchronized systems (large coupling strength).

In order to avoid misinterpretations about directionality in regimes of zero and strong couplings, in the following we consider—for each combination of pairs of electrode contacts—only those values of *d* and *T*, for which the accompanying values of the strength of interactions (*R* and *γ*) are from the interquartile range of the respective distribution of these values^35^,^36^.

To test for possible influences due to various physiologic and pathophysiologic processes, we performed additional analyses using subsets of the data:

- subset *day* includes all data from daytimes (6 am to 10 pm);
- subset *night* includes all data from night times (10 pm to 6 am; since no sleep-scoring was available for the patients investigated here, we cannot evaluate the influence of different sleep stages);
- subset *pre-ictal* includes all data from a presumed pre-ictal phase of 4 h duration^44^;
- subset *inter-ictal* includes all data from the seizure-free interval (data from the presumed pre-ictal phase, the ictal and the post-ictal phase (30 min duration) of all seizures were excluded).

We calculated—for each combination of pairs of electrode contacts—temporal averages of estimators for the strength of interactions (denoted as 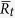 and 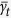), either for the aforementioned subsets or for the full dataset of a given patient. With the restrictions for the estimators for the direction of interactions outlined above, we counted the number of windows with a specific indication for directionality, i.e., either (*d* > 0, *T* > 0) or (*d* < 0, *T* < 0). The cases of independence or bidirectional interactions (*d* = 0, *T* = 0) were not considered here. With Δ^PA^ and Δ^IA^, we denote the rate of a specific directionality indication as the number of windows, for which a given estimator indicates this specific directionality divided by the total number of windows with an identifiable directionality. With the asymmetry of estimators under the exchange of *X* and *Y*, we define (Δ^PA^, Δ^IA^) > *ϑ* to indicate directed interactions to be driving more frequently, and (Δ^PA^, Δ^IA^) < *ϑ* to indicate directed interactions to be responding more frequently (*ϑ* = 0.5 indicates an equal rate of driving and responding indications). Eventually, we averaged the estimators for the strength of interaction *R* and *T* as well as the rates Δ^PA^ and Δ^IA^ over all combination of pairs of electrode contacts for a given pair of brain regions. These mean values will be denoted as 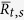, 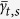, 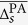, and 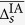 in the following.

To quantify to which extent the IA- and PA-based spatially averaged rates of a specific directionality indication 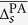 and 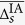 exceed what would be expected for chance, we compared these rates against a null distribution of rates that we derived from a random assignment of pairs of electrodes to a given interaction within or between brain regions (thereby preserving the initial population density). For each directionality analysis approach, we performed twenty assignment runs for each patient and each interaction, calculated the mean rate and its standard deviation, and with the mean rate plus/minus the standard deviation we derived the range [*ϑ*^−^, *ϑ*^+^] of rates above/below which we define a rate as an indication for a *predominant* driving/responding.

### Exploratory statistical analyses

#### Testing for differences in interactions within and between brain regions

We applied Mann–Whitney *U* tests to probe for possible differences in interactions across patients using either the full datasets or the aforementioned subsets. We investigated the temporally and spatially averaged strength (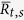 and 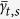) of all interactions (*f*–*f, f*–*n, f*–*o, n*–*n, n*–*o, o*–*o*) as well as the spatially averaged rates 
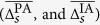
 between (but not within) brain regions (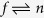, 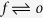, 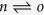) and considered differences significant at the *p* = 0.05 significance level (one-sided tests; Bonferroni correction for multiple testings). In addition, we performed hypergeometric tests to check whether possible differences may result from the strongly varying number of pairs of electrode contacts that contribute to interactions within and between brain regions (cf. Table 3). To this end, we drew (without replacement), for each patient, from the set of all interaction strengths the highest values of 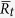, respectively 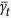, identified the associated brain regions, and estimated the hypergeometric distributions for these regions. We performed the same analysis with the set of the rates of a specific directionality indication Δ^PA^, respectively Δ^IA^.

We report the number of patients for which the twenty highest strengths of interactions respectively the twenty highest rates of a specific directionality indication could be observed significantly (*p* < 0.05) more often than what would be expected by chance for specific interactions.

#### Testing for correlations between phase-dynamics-based and information-content-based estimators

We applied different analysis techniques to assess the extent to which the phase-dynamics-based and information-content-based estimators carry independent and non-redundant information about interaction properties. All analyses were carried out for each patient and each combination of pairs of electrode contacts, and in the following, we report mean values of the extend over all pairs of electrode contacts that contributed to interactions within and between brain regions obtained from either the aforementioned subsets or for the full dataset of a given patient. For the strength of interactions, we calculated the Pearson product-moment correlation coefficient *ρ* between the temporal evolutions of *R* and *γ* and considered only those values of *ρ* with a significance level of *p* < 0.05 after Bonferroni correction.

With the restriction for the estimators for the direction (see above), we calculated—using Allen’s Interval Algebra^70^—the relative amount of time 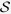 with both PA and IA indicating the same directionality (driving, responding, bidirectional). We here only considered Allen’s base relation *a* = *b* to assess 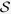.

## Additional Information

**How to cite this article**: Dickten, H. *et al*. Weighted and directed interactions in evolving large-scale epileptic brain networks. *Sci. Rep.* **6**, 34824; doi: 10.1038/srep34824 (2016).

## Figures and Tables

**Table 1:** Number (percentage) of patients for which the twenty highest strengths of interactions (see Methods) could be observed significantly (*p* < 0.05, hypergeometric test) more often than by chance in a given interaction (note that a patient may contribute more than once to each interaction).

Approach	Interactions					
	*f*–*f*	*f*–*n*	*f*–*o*	*n*–*n*	*n*–*o*	*o*–*o*
PA	21 (62%)	3 (10%)	1 (3%)	6 (24%)	1 (3%)	13 (37%)
IA	22 (65%)	3 (10%)	1 (3%)	6 (24%)	0 (0%)	12 (34%)

**Table 2:** Number (percentage) of patients for which the twenty highest rates of a specific directionality indication could be observed significantly (*p* < 0.05, hypergeometric test) more often than by chance in a given interaction (note that a patient may contribute more than onces to each interaction).

Approach	Interactions					
	*f* → *n*	*f* → *o*	*n* → *f*	*n* → *o*	*o* → *f*	*o* → *n*
PA	3 (10%)	24 (69%)	3 (10%)	8 (27%)	6 (17%)	5 (17%)
IA	4 (13%)	16 (46%)	2 (7%)	11 (37%)	6 (17%)	5 (17%)

**Table 3:** Mean number of pairs of electrode contacts for interactions within and between brain regions.

Interactions						Total
*f*–*f*	*f*–*n*	*f*–*o*	*n*–*n*	*n*–*o*	*o*–*o*	
63.3	48.1	327.1	13.2	161.8	830.0	1513.4
