# Erratum: Weighted and directed interactions in evolving large-scale epileptic brain networks

**DOI:** 10.1038/srep38272

**Published:** 2016-12-09

**Authors:** Henning Dickten, Stephan Porz, Christian E. Elger, Klaus Lehnertz

Scientific Reports 6: Article number: 3482410.1038/srep34824; published online: 10 06 2016; updated: 12 09 2016

The Acknowledgments section in this Article is incomplete:

“We are grateful to Gerrit Ansmann and Christian Geier for interesting discussions and for critical comments on earlier versions of the manuscript and by the Verein zur Foerderung der Epilepsieforschung e.V. (Bonn)”.

should read:

“We are grateful to Gerrit Ansmann and Christian Geier for interesting discussions and for critical comments on earlier versions of the manuscript. This study was supported by the Deutsche Forschungsgemeinschaft DFG (Grant No.: LE 660/5-2) and by the Verein zur Foerderung der Epilepsieforschung e.V. (Bonn)”.

